# Machine Learning Model for Anesthetic Risk Stratification for Gynecologic and Obstetric Patients: Cross-Sectional Study Outlining a Novel Approach for Early Detection

**DOI:** 10.2196/54097

**Published:** 2024-08-21

**Authors:** Feng-Fang Tsai, Yung-Chun Chang, Yu-Wen Chiu, Bor-Ching Sheu, Min-Huei Hsu, Huei-Ming Yeh

**Affiliations:** 1 Department of Anesthesiology National Taiwan University Hospital Taipei Taiwan; 2 Graduate Institute of Data Science Taipei Medical University Taipei Taiwan; 3 Clinical Big Data Research Center Taipei Medical University Hospital Taipei Taiwan; 4 Clinical Data Center Office of Data Science Taipei Medical University Taipei Taiwan; 5 Medical Research Department College of Medicine National Taiwan University Taipei Taiwan; 6 Department of Obstetrics and Gynecology College of Medicine National Taiwan University Taipei Taiwan

**Keywords:** gradient boosting machine, comorbidity, gynecological and obstetric procedure, ASA classification, American Society of Anesthesiologists, preoperative evaluation, machine learning, machine learning model, gynecology, obstetrics, early detection, artificial intelligence, physiological, gestational, anesthetic risk, clinical laboratory data, laboratory data, risk, risk classification

## Abstract

**Background:**

Preoperative evaluation is important, and this study explored the application of machine learning methods for anesthetic risk classification and the evaluation of the contributions of various factors. To minimize the effects of confounding variables during model training, we used a homogenous group with similar physiological states and ages undergoing similar pelvic organ–related procedures not involving malignancies.

**Objective:**

Data on women of reproductive age (age 20-50 years) who underwent gestational or gynecological surgery between January 1, 2017, and December 31, 2021, were obtained from the National Taiwan University Hospital Integrated Medical Database.

**Methods:**

We first performed an exploratory analysis and selected key features. We then performed data preprocessing to acquire relevant features related to preoperative examination. To further enhance predictive performance, we used the log-likelihood ratio algorithm to generate comorbidity patterns. Finally, we input the processed features into the light gradient boosting machine (LightGBM) model for training and subsequent prediction.

**Results:**

A total of 10,892 patients were included. Within this data set, 9893 patients were classified as having low anesthetic risk (American Society of Anesthesiologists physical status score of 1-2), and 999 patients were classified as having high anesthetic risk (American Society of Anesthesiologists physical status score of >2). The area under the receiver operating characteristic curve of the proposed model was 0.6831.

**Conclusions:**

By combining comorbidity information and clinical laboratory data, our methodology based on the LightGBM model provides more accurate predictions for anesthetic risk classification.

**Trial Registration:**

Research Ethics Committee of the National Taiwan University Hospital 202204010RINB; https://www.ntuh.gov.tw/RECO/Index.action

## Introduction

Evaluating perioperative risk is an important part of preoperative assessment [1]. The American Society of Anesthesiologists (ASA) physical status classification system is used to assess a patient’s medical conditions before anesthetic induction. A healthy patient with well-controlled disease can be classified as ASA class I or II, indicating low anesthetic risk, whereas a patient with impaired organ function is classified as ASA class III or higher, indicating high anesthetic risk. ASA physical status scores are correlated with the risk of postoperative complications, particularly the risk of mortality. Effective risk prediction is the key to optimizing patient care and resource allocation in health care settings. Patients with high anesthetic risk require more intensive postanesthetic care and longer hospital stays than those with low anesthetic risk [2]. The ASA scoring process is not straightforward. The score is calculated based on the experience of anesthesiologists, who make assessments according to the status of organ function [3,4]. Only anesthesiologists with years of experience can effectively integrate all coexisting issues into an ASA classification [5]. Assessments include laboratory data, comorbidities, and the specific procedure. Several machine learning programs are available; however, it is a struggle to apply the findings to clinical practice. Only big data analytics can reveal the interaction between patient organ function and anesthetic risk [6,7].

Advances in artificial intelligence have been made in various fields, including anesthesiology. Machine learning can be integrated into intraoperative anesthetic practice and can be applied for preoperative ASA prediction. Several research groups have attempted to train a machine learning algorithm for ASA physical status classification; however, in most cases, physicians or specialists are still required for evaluation [8,9]. Although models in several studies have achieved high accuracy, they have failed to address the class imbalance between ASA physical status classes, which can skew results. In one study, the ASA physical status scores for all surgeries were evaluated by a single anesthesiologist, who concluded that the ICD-9 (*International Classification of Diseases, Ninth Revision*) was the most significant contributor; however, selection bias cannot be excluded [10]. This study explored the application of machine learning methods for anesthetic risk classification and evaluated the contributing factors in clinical practice. To minimize the effects of confounding variables during training, we used a homogenous group with similar physiological states and ages undergoing similar pelvic organ procedures not involving malignancies. We selected patients from the gynecologic and obstetric wards based on gestation age because this provided the most uniform criterion apart from gestation itself. We used machine learning for ASA classification and for evaluating the contributions of the ICD-10 (*International and Statistical Classification of Diseases, Tenth Revision*).

In this study, we developed a predictive methodology based on a light gradient boosting machine (LightGBM) model for anesthetic risk stratification for gynecologic and obstetric patients. Our research has several key features. First, we used machine learning methods that can analyze large amounts of clinical data that can identify patterns and learn relationships—an approach not commonly used in gynecologic and obstetric anesthetic risk classification. Second, we incorporated comorbidity information and clinical laboratory data into our model. Comorbidity information reflects additional diseases or health issues that patients may develop during anesthesia induction and is a crucial component of anesthetic risk assessment. Clinical laboratory data includes physiological indicators and pathological characteristics, which enable a more comprehensive evaluation of anesthetic risk. By integrating these two types of information into our model, we enhanced the model’s accuracy and predictive capabilities. Finally, we focused on model visualization and interpretability by explaining predictions through visual input-output representation and by ranking the importance of key features. These analyses can help physicians and clinical anesthetists better understand the working principles of the model and can provide valuable clinical insights for implementing improved anesthesia strategies and decision-making.

## Methods

### Overview

**Figure 1:**
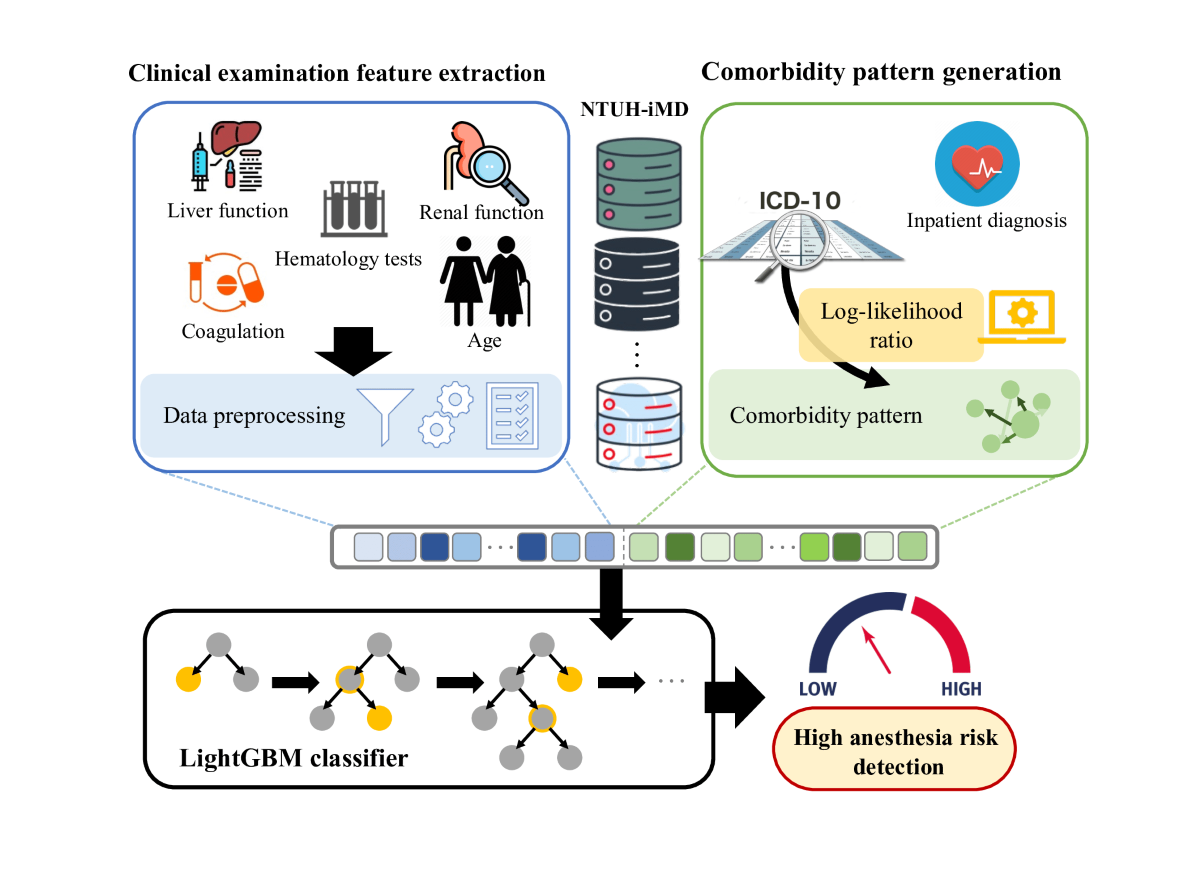
Proposed model system architecture. ICD-10: International Classification of Diseases, 10th Revision; LightGBM: light gradient boosting machine; NTUH-iMD: National Taiwan University Hospital Integrated Medical Database.

In this section, we present our method for automatically detecting patients with high anesthetic risk. The system architecture, which is illustrated in Figure 1, consists of 4 key components: National Taiwan University Hospital Integrated Medical Database (NTUH-iMD), clinical examination feature extraction, comorbidity pattern generation, and LightGBM. First, the clinical examination feature extraction component retrieves clinical examination data from the NTUH-iMD and performs data preprocessing to generate a clinical examination feature vector. Next, the comorbidity pattern generation component accesses inpatient diagnostic data from the NTUH-iMD and uses *ICD-10* codes to identify comorbidities and then generate comorbidity patterns as comorbidity feature vectors. Finally, the 2 generated vectors are merged as input for the LightGBM component. Thus, a classifier can be trained to detect patients with high anesthetic risk.

### Ethical Considerations

In this study, the inclusion criteria were female patients of reproductive age (age 20-50 years) who underwent gestational or gynecological surgery between January 1, 2017, and December 31, 2021, which were obtained from the NTUH-iMD. This study was registered with the Research Ethics Committee of the National Taiwan University Hospital (202204010RINB). The informed consent waiver was also approved by the same Research Ethics Committee. All the data of this study were deidentified and confidentiality protected with file system level encryption. There is no identification of individual participants or users in any images of the paper or supplementary materials.

### NTUH-iMD Data Set

Data on women of reproductive age (age 20-50 years) who underwent gestational or gynecological surgery between January 1, 2017, and December 31, 2021, were obtained from the NTUH-iMD. Most of the hospitalized patients with benign gynecological procedures and patients with all gestational operations were included. Patients requiring malignant-related procedures were excluded. Our variables encompassed patient demographic information such as age, surgery time, hospital level, comorbidities, pharmacy prescriptions, outpatient visits, emergency room visits, and hospitalization. Additionally, we collected data on medication use including cardiovascular and renal drugs, endocrine and metabolic drugs, respiratory tract drugs, hematologic drugs, endocrine drugs, and anti-infective agents. A total of 10,946 patients were identified in the preliminary group. Among these patients, complete data were unavailable for 54 patients, who were subsequently excluded. Finally, we included 10,892 patients in the analysis. Within our data set, 9893 patients were classified as having low anesthetic risk (ASA 1-2), and 999 patients were classified as having high anesthetic risk (ASA >2). There were 4532 (41.6%) inclusions received gestation-related, mainly cesarean section; 6360 (58.4%) inclusions received gynecological surgeries.

### Clinical Examination Feature Extraction

Medical examinations and anesthetic risk are inherently interconnected. We identified the 15 most frequently conducted assessment items prior to surgery and used these items as the features in our research model [11]. These items can be categorized into 5 major domains of examination: hematology tests, renal function, coagulation, liver function, and others. Hematology tests encompass several parameters related to different aspects of blood composition. Red blood cell (RBC) count, the quantity of RBCs in a given blood volume, indicates the blood’s oxygen-carrying capacity and can be used to identify anemia. Hemoglobin, a protein molecule found in RBCs, reflects the blood’s oxygen-carrying capacity and aids in identifying anemia. Hematocrit is the concentration of RBCs in the blood, aiding in evaluating the blood’s oxygen-carrying capacity and in identifying dehydration and polycythemia. Mean corpuscular hemoglobin denotes the average amount of hemoglobin within each RBC, assisting in assessing the blood’s oxygen-carrying capacity and identifying specific types of anemia. Mean corpuscular hemoglobin concentration, the average concentration of hemoglobin in a given volume of packed RBCs, aids in evaluating the color and concentration of hemoglobin and in identifying anemia. Mean corpuscular volume can be used to measure the average size or volume of RBCs, which helps categorize anemia as microcytic, normocytic, or macrocytic and assists in identifying underlying causes. White blood cell (WBC) count, the total number of WBCs in a given blood volume, provides an assessment of immune system function, identification of infections, and monitoring of the treatment response. Platelet count, the number of platelets in a given blood volume, can be used to evaluate the blood’s clotting ability and to identify and monitor conditions such as thrombocytopenia or thrombocytosis. Finally, the red cell distribution width coefficient of variation measures the variation in the size of RBCs, aiding in the diagnosis of different types of anemia and monitoring the treatment response.

Renal function tests are used to evaluate kidney function. One of the key measures is the estimated glomerular filtration rate (EGFR), which calculates the rate at which the kidneys filter waste products from the blood. The EGFR is used to assess kidney function and diagnose or monitor conditions such as chronic kidney disease. Another important parameter is creatinine, a waste product produced by muscle metabolism that is filtered by the kidneys. Measuring the creatinine concentration helps evaluate kidney function and diagnose or monitor conditions such as kidney disease. These renal function parameters are crucial for assessing the health and functioning of the kidneys. In addition, coagulation tests are used to evaluate the blood’s clotting ability. The prothrombin time (PT) test measures the time it takes for blood to clot and is used to assess the activity of clotting factors in the blood and monitor anticoagulant therapy. The activated partial thromboplastin time test also measures the clotting time, specifically assessing the intrinsic pathway of coagulation. This test is used to monitor anticoagulant therapy and diagnose bleeding disorders. Additionally, the PT international normalized ratio, a standardized measure derived from the PT test, is used to monitor the effectiveness of anticoagulant therapy and assess the risk of abnormal bleeding. These coagulation tests play a crucial role in evaluating clotting function and in guiding treatment decisions. The last feature set is related to liver function. Liver function tests are performed to assess the health and function of the liver. These tests involve the measurement of various liver enzymes, which provide insights into liver health. One such enzyme is aspartate aminotransferase (AST), which is primarily found in the liver, heart, and skeletal muscles. Measuring AST levels aids in evaluating liver function and in identifying liver disease or damage. AST is an important marker for assessing the overall health of the liver and for identifying potential liver-related issues.

In addition to the aforementioned feature sets, age was included to explore its effect on anesthetic risk. Finally, 16 comprehensive features were included in our model. These features encompass various domains, namely hematologic parameters (RBC, hemoglobin, hematocrit, mean corpuscular hemoglobin, mean corpuscular hemoglobin concentration, mean corpuscular volume, WBC, platelet, red cell distribution width-coefficient of variation), renal function tests (EGFR and creatinine), coagulation tests (PT, activated partial thromboplastin time, and PT international normalized ratio, liver function (AST), and patient age. These features provide a robust foundation for the development of our model, enabling analysis in their respective domains. It is worth noting that the range of values for clinical examination and comorbidity varies significantly, which could potentially negatively impact the performance of a machine-learning model. To address this issue, we have implemented normalization procedures using the *z* score method for continuous variables. This ensures that each feature contributes equally to the model’s learning process. Such an approach is effective in mitigating any adverse effects arising from differences in scale among the variables.

### Comorbidity-Integrated LightGBM Model for Predicting Risk of Gynecological and Obstetric Anesthesia

Comorbidity refers to the simultaneous presence of two or more diseases, which typically affects the overall health of a patient. Given the potential correlation and interaction between comorbidities and anesthetic risk in clinical practice, this study incorporated comorbidities into the model to comprehensively assess the overall risk in patients and provide more accurate predictions of anesthetic risk. We used the log-likelihood ratio (LLR), which is an effective feature selection method that can generate representative comorbidities in patients with high anesthetic risk [12]:



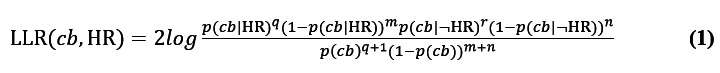



Using a training data set comprising binary labels indicating whether patients were identified as high risk (HR) or not high risk (¬HR), we obtained primary and secondary diagnoses from inpatient records to generate a set of co-occurring diseases, representing comorbidities as {*cb_1_*,..., *cb_n_*}. The LLR uses a specific mechanism to calculate the probability that co-occurring diseases in high-risk patients are not a result of chance. To illustrate this calculation, consider a specific comorbidity. *N*(HR) and *N*(*¬*HR) represent the numbers of high-risk and not-high-risk patients, respectively. *N*(*cb^*HR), denoted as *q*, indicates the number of high-risk patients with comorbidity *cb*. By contrast, *N*(*cb^¬*HR), denoted as *r*, represents the number of not-high-risk patients with comorbidity *cb*. To simplify the formula, we defined *m* as *N*(HR) – *q*, which represents the number of high-risk patients without comorbidity *cb*, and *n* as *N*(*¬*HR) – *r*, which denotes the number of not-high-risk patients without comorbidity *cb*. A maximum likelihood estimation is then performed to derive the probabilities *p*(*cb*), *p*(*cb|*HR), and *p*(*cb|^*HR) by calculating the LLR of the hypothesis that the presence of *cb* in the high-risk patient set is not random. A large LLR value for co-occurring diseases suggests a strong association with high anesthetic risk. The training data are used to rank all disease pairs based on their respective LLR values. We selected disease pairs with high scores as representative of comorbidity (ie, comorbidity patterns) related to anesthesia.

Once the comorbidity representation was obtained, we integrated it with clinical examination features through the direct splicing strategy to form a consolidated feature vector as the input to the model. In this study, LightGBM was used as the classification model training algorithm [13]. LightGBM is a highly efficient and accurate machine learning method widely used in various data modeling and prediction tasks. It operates based on the principles of gradient boosting, in which multiple weak learners are sequentially added to enhance the model’s performance. LightGBM is distinct from traditional gradient-boosting machines in terms of its unique operational principles. It uses a histogram-based decision tree (DT) algorithm that leverages the binning of feature values and histogram-based sparse feature optimization. These techniques improve training speed and memory use efficiency. Additionally, LightGBM incorporates exclusive feature bundling and gradient-based one-side sampling to further accelerate the training process. LightGBM has been extensively applied across various machine learning tasks including classification, regression, ranking, and recommendation systems. Consequently, the model is widely used in data competitions and real-world applications [14-16].

### Comparative Analysis Models

We conducted a comprehensive comparative analysis of several widely used predictive models to assess their suitability for anesthetic risk stratification. We selected a set of well-known machine-learning approaches as the baseline for our evaluation. The first model considered was Naïve Bayes (NB), a probabilistic classifier that applies Bayes’ theorem. The NB classifier assumes independence between features and is computationally efficient. The second model was logistic regression (LR), a linear classifier that models the relationship between independent variables and the log odds of the dependent variable. LR is widely used in medical research due to its interpretability and ability to analyze both categorical and continuous variables. We also included the k-nearest neighbor (KNN) algorithm, a nonparametric method that classifies instances based on their proximity to labeled instances in the training set. The KNN algorithm is flexible and can analyze various types of data, making it suitable for anesthetic risk stratification. Another model in our comparison was the DT, a tree-based model that creates decision rules based on feature thresholds. DTs offer interpretability and can capture nonlinear relationships, which are crucial for understanding the underlying factors influencing anesthetic risk. Finally, we considered the support vector machine (SVM), a binary classifier that finds an optimal hyperplane to separate data into different classes. SVMs are particularly effective for high-dimensional data and can capture nonlinear relationships using kernel functions.

In addition to the aforementioned models, we considered 3 other prominent methods for our comparative analysis: random forest (RF), extreme gradient boosting (XGBoost), LightGBM, and multilayer perceptron (MLP). RF is an ensemble learning method that combines multiple DTs to make predictions. It addresses issues, such as overfitting and instability, by aggregating the predictions of individual trees. RF is robust and is known for its ability to examine high-dimensional data. Additionally, it provides valuable insights through feature importance measures, which help identify the most influential variables in the anesthetic risk stratification process. XGBoost [17], another ensemble learning method, uses gradient boosting to construct a powerful predictive model. It is known for its computational efficiency and ability to address missing data effectively. XGBoost also provides feature importance measures, allowing researchers to understand the relative contributions of different features to the anesthetic risk stratification process. To improve our proposed method, we incorporated LightGBM, which is a high-performance gradient-boosting framework that excels in examining large-scale data sets. Its efficient tree-based learning algorithm enables rapid training and prediction. With optimized memory use and excellent parallelization, LightGBM excels in complex machine learning and data analysis tasks, making it a powerful tool for addressing real-world challenges. Finally, we included the MLP [18], a neural network inspired by the structure and function of the human brain. MLP can capture complex relationships and patterns in data, making it well-suited for tasks involving intricate interactions. MLP excels at learning from large data sets, which is advantageous for anesthetic risk stratification, in which a comprehensive understanding of the patient’s medical history is crucial.

Through the evaluation of these models, our objective was to determine an optimal approach for precise anesthetic risk stratification, considering interpretability, computational efficiency, and capturing complex relationships.

### Evaluation Data Set and Experimental Settings

To ensure the reliability of our experimental results, a 10-fold cross-validation approach was conducted. This approach is widely used in research to assess the performance and generalizability of machine learning models. In 10-fold cross-validation [12], the data set is divided into 10 subsets of approximately equal size. The training and evaluation process is then performed 10 times, with each fold serving as the validation set while the remaining 9 folds are used for training. By rotating the validation set across all 10 folds, we obtain a more comprehensive evaluation of our model’s performance. This rigorous validation technique mitigates the effects of random variations and provides a robust assessment of the effectiveness of our approach. Furthermore, to address the class imbalance issue commonly found in medical data sets, we used the synthetic minority oversampling technique for effective resampling. The synthetic minority oversampling technique operates by randomly selecting an instance from the minority class and identifying its KNNs within the same class. A synthetic instance is then generated by randomly selecting one of these neighbors, and a line segment is formed between the selected instance and the neighbor in the feature space. These synthetic instances are created as a combination of the 2 chosen instances, a and b, ensuring convexity. By using the synthetic minority oversampling technique, we balanced the data distribution between positive and negative instances. In our specific experiment, we oversampled clinical narratives, vital signs, and patient demographic data from the minority classes to achieve this balance [19].

In our experimental settings, we implemented an MLP comprising 3 fully connected layers. The configuration begins with an input layer featuring neurons equal in number to the input features, followed by an intermediary layer where the neuron count is halved. The architecture culminates in an output layer with a single neuron, designed to represent the probability of HR. The activation function used throughout is rectified linear unit, a type commonly used in the hidden layers of neural networks. Additionally, the loss function used is binary cross entropy. Optimization was carried out using the Adam optimizer, set to a learning rate of 0.001. For our SVM model, we chose a radial basis function kernel. Furthermore, we used LightGBM with the following parameter configurations to optimize its performance for our specific task. The “*gbdt*” boosting type uses gradient boosting with DTs, and the choice of 31 leaves provides a balance between model complexity and generalization. Setting the maximum depth to –1 allows the trees to grow without any restrictions on depth. A learning rate of 0.1 controls the contribution of each tree in the ensemble. We trained the model with 100 estimators to capture sufficient complexity and avoid overfitting. Additionally, we assigned the class weights of 0.1 and 0.9 to the minority (0) and majority (1) classes, respectively, to address any class imbalance present in the data set.

The performance of our model for predicting gynecological and obstetric mortality was evaluated using precision, recall, and *F*_1_-score metrics. Additionally, microaveraged metrics were used to assess the overall performance of the models. These evaluation measures were determined based on a contingency table that captured the predictions for a specific target criterion, *C_i_*. Precision (*P*(*C_i_*)), recall (*R*(*C_i_*)), and *F*_1_-score (*F*_1_(*C_i_*)) were calculated as follows:



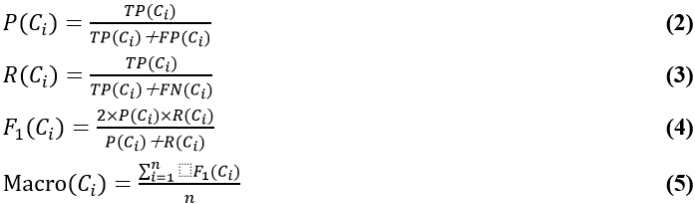



Here, *TP*(*C_i_*) represents the number of true positives, which are instances correctly classified as positive, and *FP*(*C_i_*) represents the number of false positives, which are negative instances mistakenly classified as positive. Similarly, *TN*(*C_i_*) and *FN*(*C_i_*) denote the number of true negatives and false negatives, respectively. The *F*_1_-score provides a comprehensive assessment of the relative effectiveness of the compared methods. As mentioned in the NTUH-iMD Data Set section, the data set contained fewer high-risk than low-risk patients. The ratio of high-risk to low-risk patients was approximately 10:1. A macroaveraging (Macro (*C_i_*)) approach was used to calculate the overall performance of each model, allowing for a more comprehensive and objective evaluation of each model’s performance.

In addition to precision and recall, sensitivity and specificity were applied as evaluation metrics to analyze the performance of our model. Sensitivity measures the proportion of correctly identified positive instances, reflecting the model’s ability to accurately detect true positives. Specificity quantifies the proportion of correctly identified negative instances, indicating the model’s ability to correctly identify true negatives. These metrics provide insights into the model’s overall accuracy in identifying positive and negative instances. To visually assess the performance of our model and quantify its discriminatory power, we used the receiver operating characteristic curve. The receiver operating characteristic curve illustrates the trade-off between true positive rate (sensitivity) and false positive rate (1 – specificity) at various classification thresholds. This curve provides a visual representation of the model’s performance across a range of classification thresholds. To further quantify discriminatory ability, we used the area under the receiver operating characteristic curve as a performance metric. The area under the receiver operating characteristic curve provides a single scalar value that measures the overall performance of the model. A higher value for the area under the receiver operating characteristic curve indicates that the model more effectively and accurately distinguishes between positive and negative instances.

## Results

**Figure 2:**
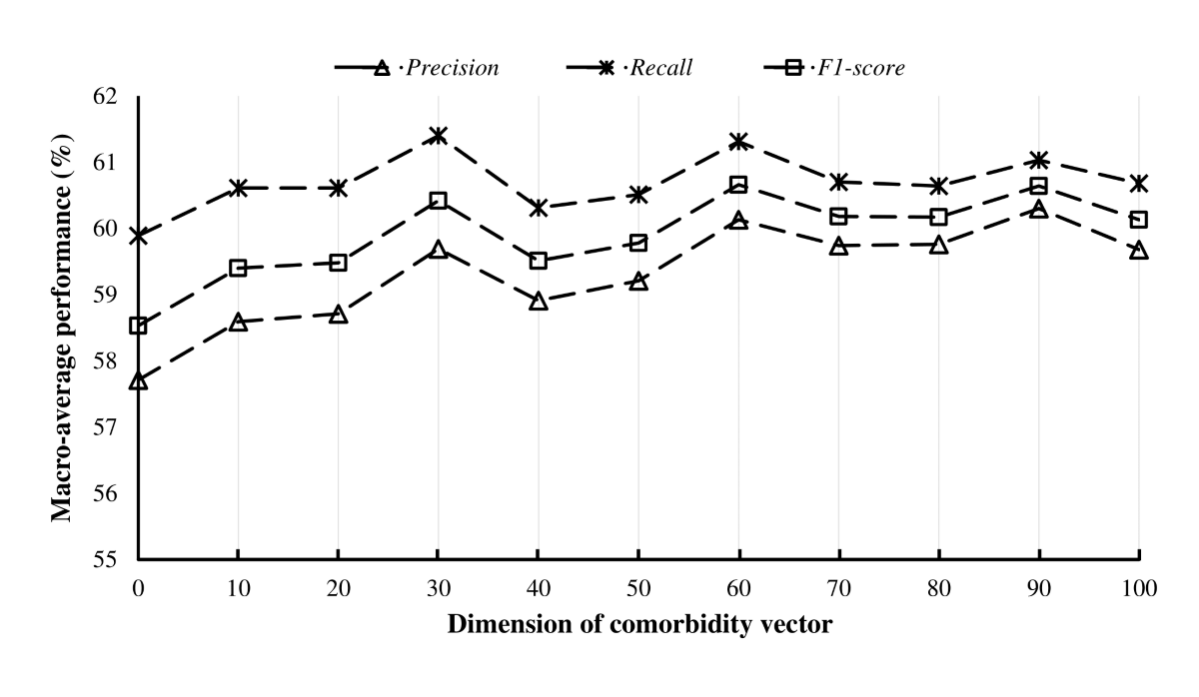
Precision-recall-*F*^1^-score curve with different dimensions of the comorbidity vector.

Our approach incorporated a single parameter *ζ*, which represents the number of comorbidity patterns used for data representation. To investigate the effect of this parameter, we conducted experiments by varying *ζ* from 0 to 100 in increments of 10. The model’s predictive performance under different parameter settings is illustrated in Figure 2. Precision and recall rates were generally positively correlated with *ζ*. This can be attributed to the fact that a higher value of *z* allows our model to consider a larger number of comorbidity patterns that are strongly associated with high anesthetic risk. Consequently, this setting enhances the detection of high anesthetic risk. Optimal detection performance was achieved when *ζ* was set to 60 (Figure 2). Accordingly, this value was used in subsequent evaluations.

**Figure 3:**
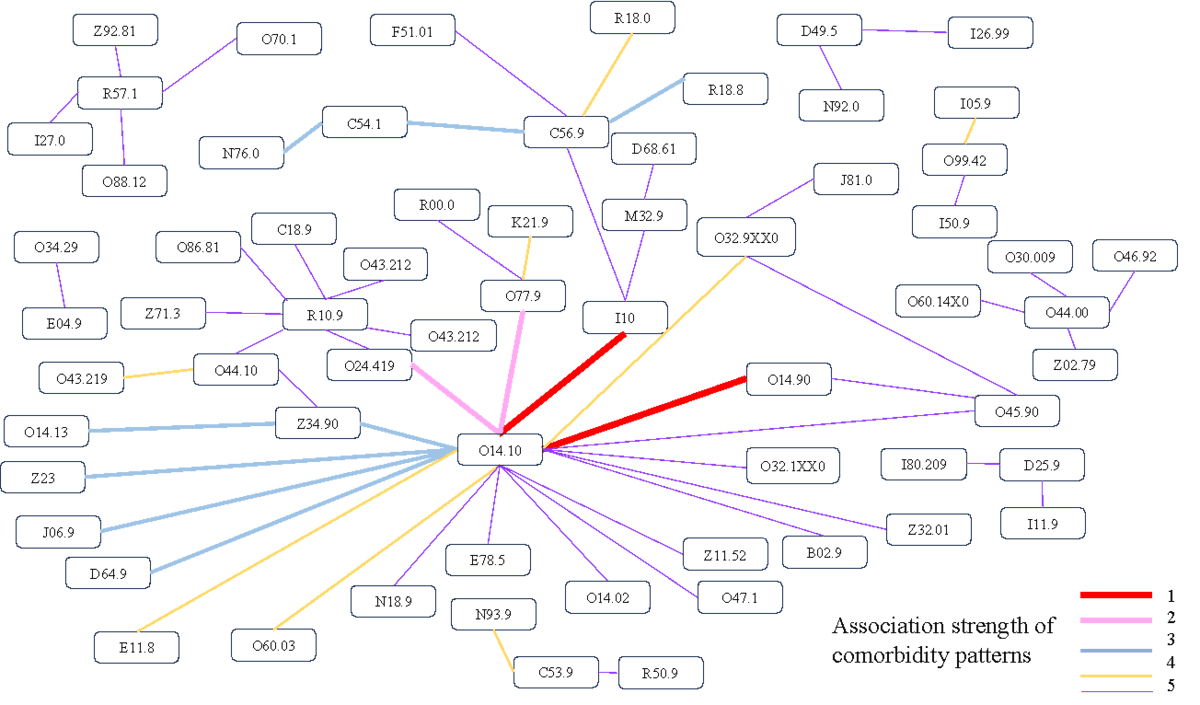
Network visualization of comorbidities associated with anesthesia risk.

As shown in Figure 3, the network visualization illustrates the comorbidity patterns associated with anesthesia risk, generated from our study’s data. The thickness of the lines represents varying degrees of association strength between comorbidities, quantified by their respective LLR values. We have categorized these associations into 5 levels of strength, with level 1 being the strongest and level 5 the weakest, each denoted by different colors for clarity. It is worth noting that O14.10 (severe preeclampsia, unspecified trimester) is the most important component of comorbidity patterns, which is linked to many other diseases, indicating a strong association with increased anesthesia risk. Based on clinical evidence, this suggests that severe preeclampsia increases the risk of anesthesia-related complications during surgery. This visualization serves not only as a tool for visualizing data but also as a critical aid in understanding potential implications in clinical practice.

We further compared some well-known machine learning, ensemble learning, and deep learning models for comprehensive analysis (Table 1). As a benchmark method, the DT model exhibited the lowest performance among all models evaluated. Its performance on all metrics was less than satisfactory, possibly due to the model’s simplicity, which renders it unable to efficiently capture the intricacy of the data. The NB classifier demonstrated intermediate overall performance. Despite its precision and recall not being particularly high, it maintained a relatively balanced equilibrium between these 2 metrics. This suggests that the NB classifier can regulate false positives and false negatives with equal efficacy. The precision of LR was on par with that of NB, but it showed higher recall, indicating that it identified a larger number of true positive cases. The SVM model demonstrated moderate performance in our anesthesia risk assessment, achieving precision, recall, and *F*_1_-scores of 56.77%, 57.80%, and 57.28%, respectively. It uses a hyperplane for data classification and excels in managing nonlinear boundaries through the application of a radial basis function kernel. A high-dimensional medical data set, like those in anesthesia risk, has numerous variables and overlapping classes, making this capability especially useful. SVM was able to balance precision and recall effectively, ensuring a reliable level of accuracy.

**Table 1:** Performance of compared predictive models.

ML^a^ methods	*P*/*R*/*F*_1_ (%)	Ensemble and DL^b^ methods	*P*/*R*/*F*_1_ (%)
NB^c^	55.16/58.75/56.90	RF^d^	67.67/51.83/58.70
LR^e^	55.43/62.19/58.62	XGBoost^f^	58.25/56.68/57.45
KNN^g^	67.64/51.96/58.77	LGBM^h^	62.93/53.67/57.93
DT^i^	53.70/53.99/53.84	MLP^j^	65.48/52.28/58.14
SVM^k^	56.77/57.80/57.28	Our method	60.26/61.40/60.78

^a^ML: machine learning.

^b^DL: deep learning.

^c^NB: Naïve Bayes.

^d^RF: random forest.

^e^LR: logistic regression.

^f^XGBoost: extreme gradient boosting.

^g^KNN: k-nearest neighbor.

^h^LGBM: light gradient boosting machine.

^i^DT: decision tree.

^j^MLP: multilayer perceptron.

^k^SVM: support vector machine.

**Figure 4:**
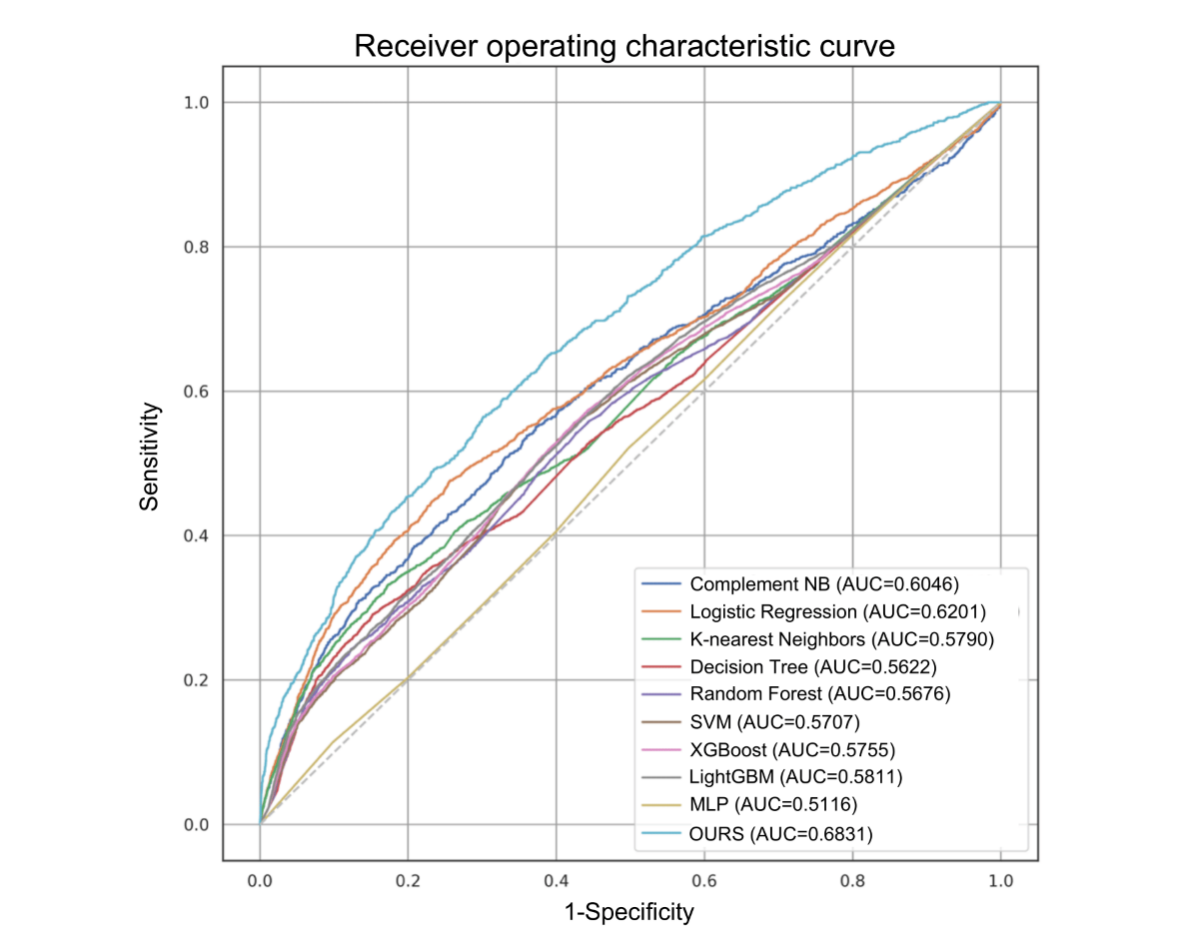
Receiver operating characteristic curve for each model. MLP: multilayer perceptron; SVM: support vector machine.

In contrast to standard machine learning methods, ensemble learning methods, such as XGBoost and LGBM, tend to deliver superior overall performance. Both methods exhibited balanced results across all metrics, with their overall efficiency achieving an *F*_1_-score of more than 57%. RF exhibited very high precision, implying a high proportion of true positives among all predicted positives. However, it had a lower recall, indicating that many true positives were missed in the process. The performance of the MLP resembled that of RF, possibly because the MLP and RF are universal function approximators that can solve complex nonlinear problems. Remarkably, our proposed method maintained a balance between precision and recall while achieving the best *F*_1_-score (60.78%), outperforming the other models. This result suggests that our method can accurately predict anesthetic risk, ensuring patient safety. Finally, we assessed the performance of the compared methods by using receiver operating characteristic curves [12]. Our method exhibited superior area under the receiver operating characteristic curve values compared with most of the compared methods (Figure 4). This finding implies that our method demonstrates a high level of accuracy for detecting high anesthetic risk.

To summarize, we evaluated several anesthetic risk prediction models. Our proposed model outperformed the other models in terms of precision, recall, *F*_1_-score, and area under the receiver operating characteristic curve. These findings highlight that our method can effectively predict anesthetic risk and enhance patient safety in medical procedures. More specifically, by incorporating comorbidity features into the model, the risk level of patients can be determined more accurately. This strategy can improve the predictive performance of the model, making it more reliable and practical and ultimately enabling more comprehensive and personalized risk assessment. This improvement can lead to better decision-making, anesthesia management, and overall health care quality for patients.

## Discussion

### Principal Findings

In line with the objectives delineated in the introduction, this study successfully developed and validated a predictive methodology using the LightGBM model for anesthetic risk stratification in gynecologic and obstetric patients. Our findings affirm the efficacy of using advanced machine learning techniques to analyze substantial clinical data sets for identifying nuanced patterns and relationships not typically discernible through conventional methods in anesthetic risk classification. Significantly, the integration of comorbidity information and clinical laboratory data enhanced the accuracy and predictive capabilities of our model by incorporating physiological indicators and pathological characteristics, thus improving the identification of patients at higher anesthetic risk. Furthermore, our methodological innovations prioritize model interpretability and visualization, enabling health care professionals to gain a deeper understanding of the predictive mechanisms at play and assisting in effective anesthesia strategy development and patient safety enhancement.

Analyzing the characteristics used in models is crucial for understanding the patterns and relationships within the data. By examining these features, we can gain insights into how the model works, identify biases, and ascertain which attributes are key to forecasting the target variable. Evaluating feature significance can reveal the relative importance of each attribute within the model and identify the attributes that considerably affect the model’s efficiency. By investigating these features, we can improve our understanding of the data and enhance the overall effectiveness of our model. Permutation feature importance is a prominent machine learning technique used to determine feature significance; in this technique, feature values are randomly shuffled, the model is retrained, and the effect of the shuffled features on performance is assessed [20]. A marked decline in performance upon shuffling suggests high importance, whereas a minimal effect indicates a low contribution. The permutation feature importance algorithm, therefore, helps identify the features with the most influence on the model’s output. The versatility of permutation feature importance enables its application across different models including LightGBM. In our research, we applied this technique to assess the predictive strength of various clinical factors.

The feature importance results were subsequently averaged. The importance of the original features under 10-fold cross-validation was evaluated using permutation feature importance (Multimedia Appendix 1). The 6 most important features were WBC, AST, EGFR, age, PT, and platelet. WBC, which indicates the number of leukocytes in your body, achieved the highest importance score (0.04). Therefore, the immune system, which defends against infection and disease, is the most important factor. AST, EGFR, age, and hematologic factors were also important. Because our data were homogenous in terms of sex and surgical type, the model focused on the main physiological difference between gynecologic and obstetric patients, which is gestation. Because WBC is commonly suspected of infection or inflammation [21], we can conclude that in our result, the WBC poses the highest risk factor of preoperative assessment [22]. Liver function and renal function undergo changes during gestation, which affects high-risk prediction [23,24]. In clinical practice, physicians and anesthetists should consider whether the complete blood count and coagulation tests are necessary components of a preoperative blood examination [25]. This model suggests that the WBC, AST, EGFR, PT, and platelet are more relevant and cost-effective factors that should be considered in the preoperative blood examination.

We further analyzed the comorbidity feature patterns generated in this study. In total, 60 comorbidity feature patterns were used in our model and divided into 5 rankings according to their LLR weighting (Multimedia Appendix 2). Notably, the comorbidities that posed the highest risk were gestation-related, with severe preeclampsia-related hypertension posing the highest risk. The second highest-ranking comorbidities were severe preeclampsia-related hyperglycemia and fetal distress. Determining ASA physical status scores for obstetric patients is not straightforward. According to the ASA Physical Status Guidelines (2020 revision), normal pregnancy is not considered a disease; yet, it is classified as ASA Physical Status Score II due to the distinct physiological state of the parturient [26]. However, little guidance is available on how to adjust for the many complications of pregnancy [27]. Obstetric anesthesiologists, when determining whether a cesarean section is necessary, consider complex gestation complications rather than age alone [28,29]. From this study, pregnant women with pathological conditions, such as those involving preeclampsia-related hypertension or hyperglycemia, should be classified as high-risk cases. Given the effect of gestation on organ function and potential threats to maternal well-being (eg, infections) or preanesthetic physical status, all abnormal conditions should be considered together.

### Limitations

This study focuses exclusively on gynecologic and obstetric patients from the National Taiwan University Hospital-Integrated Medical Database, which began recording pertinent patient assessment data prior to anesthesia in 2019. Consequently, the scope of our analysis is constrained by the duration of data availability. Additionally, as our data set predominantly comprises Taiwanese individuals, the findings may not be directly generalizable to other ethnic groups. This ethnic homogeneity limits the broader applicability of our results and underscores the need for caution when extrapolating these findings to diverse populations.

Furthermore, this research uses a binary classification framework for anesthetic risk, distinguishing only between the presence and absence of anesthesia-related risk. Anesthetic outcomes may be oversimplified with this approach, although it is useful for preliminary risk stratification. Future iterations of this research will aim to develop more nuanced models that classify anesthetic risk into multiple categories, thereby enhancing the precision of risk assessments.

### Conclusions

In conclusion, this study not only advances the application of machine learning in the field of anesthetic risk classification for gynecologic and obstetric patients but also sets a precedent for the integration of comprehensive clinical data in medical predictive models. Using the LightGBM model, our approach enhances predictive accuracy by effectively synthesizing comorbidity information with clinical laboratory data. This methodology does not merely improve anesthetic risk assessments; it facilitates a deeper understanding of the underlying factors influencing patient outcomes, thereby enabling more informed clinical decision-making. Besides its immediate clinical use, our model’s visualization and explanatory analyses expand the discourse on how machine learning can be strategically applied to enhance surgical outcomes and patient safety. These findings underscore the potential for sophisticated, data-driven approaches to transform patient care by providing anesthesiologists with precise, actionable information tailored to individual patient profiles.

In the future, we plan to incorporate a wider variety of clinical variables and patient-specific factors into our anesthetic risk stratification models. In addition to refining the models’ accuracy, we will explore their applicability across different medical fields, potentially extending beyond gynecology and obstetrics. The ultimate objective is to develop a dynamic, personalized anesthetic risk stratification framework that integrates multiple risk factors, uses advanced machine learning techniques, and leverages real-time data to substantially enhance the quality of patient care across health care settings.

## Appendix

Multimedia Appendix 1 Permutation feature importance evaluation of 16 features.

Multimedia Appendix 2 Table 2. Comorbidity feature patterns for high-risk ASA identification.

## Abbreviations

ASA: American Society of Anesthesiologists
AST: aspartate aminotransferase
DT: decision tree
EGFR: estimated glomerular filtration rate
HR: high risk
ICD-9: International Classification of Diseases, Ninth Revision
ICD-10: International and Statistical Classification of Diseases, Tenth Revision
KNN: k-nearest neighbor
LightGBM: light gradient boosting machine
LLR: log-likelihood ratio
LR: logistic regression
MLP: multilayer perceptron
NB: Naïve Bayes
NTUH-iMD: National Taiwan University Hospital Integrated Medical Database
PT: prothrombin time
RBC: red blood cell
RF: random forest
SVM: support vector machine
WBC: white blood cell
XGB: extreme gradient boosting

## Data Availability

The data sets generated during or analyzed during this study are not publicly available without permission of the National Taiwan University Hospital but are available from the corresponding author upon reasonable request.
