# Supplementary figures and images for: Machine Learning Model for Anesthetic Risk Stratification for Gynecologic and Obstetric Patients: Cross-Sectional Study Outlining a Novel Approach for Early Detection
Source: JMIR Form Res. 2024 Aug 21;8:e54097. doi: 10.2196/54097 (PMC11375379; doi:10.2196/54097)

Permutation Importance

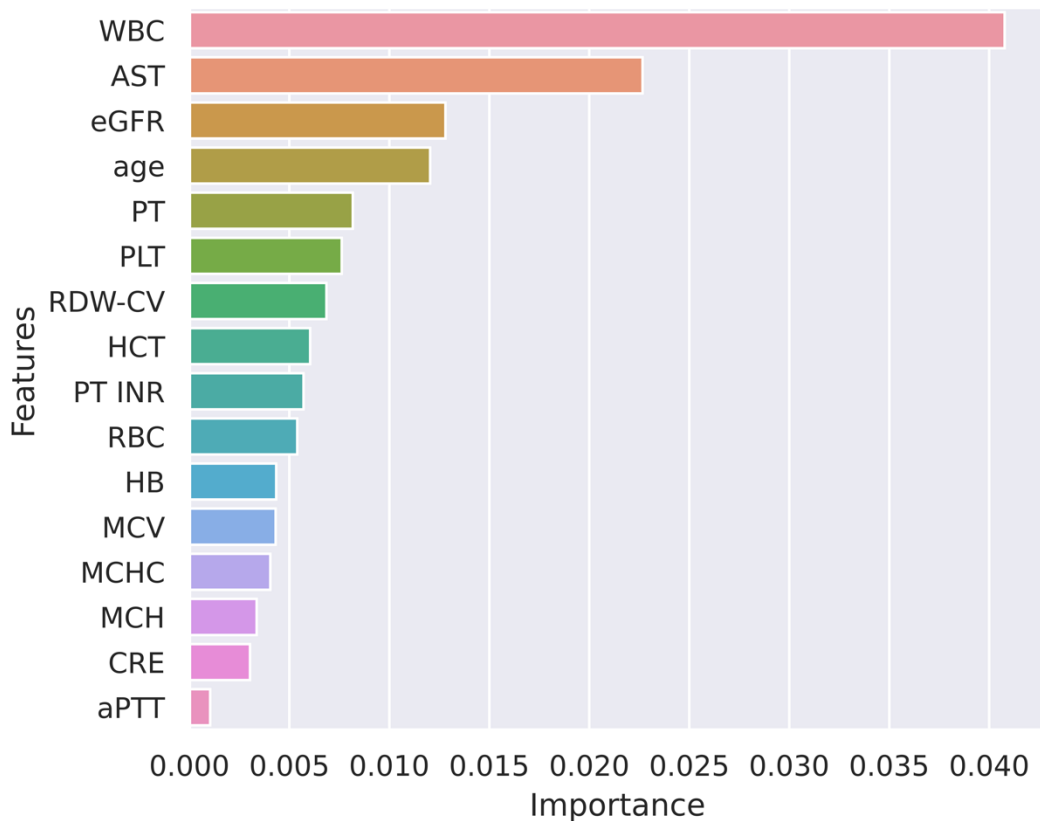

Supplement: Multimedia Appendix 1 [file formative_v8i1e54097_app1.pdf]
