# Supplementary material for: Machine Learning Model for Anesthetic Risk Stratification for Gynecologic and Obstetric Patients: Cross-Sectional Study Outlining a Novel Approach for Early Detection
Source: JMIR Form Res. 2024 Aug 21;8:e54097. doi: 10.2196/54097 (PMC11375379; doi:10.2196/54097)
Supplement: Multimedia Appendix 2 [file formative_v8i1e54097_app2.docx]

| **Rank** | **Primary diagnosis** | | **Secondary Diagnosis** | |
| --- | --- | --- | --- | --- |
|  | **Code** | **Description** | **Code** | **Description** |
| 1 | O14.10 | Severe pre-eclampsia, unspecified trimester | I10 | Essential (primary) hypertension |
|  |  |  | O14.90 | Unspecified pre-eclampsia, unspecified trimester |
| 2 | O14.10 | Severe pre-eclampsia, unspecified trimester | O24.419 | Gestational diabetes mellitus in pregnancy, unspecified control |
|  |  |  | O77.9 | Labor and delivery complicated by fetal stress, unspecified |
| 3 | C54.1 | Malignant neoplasm of endometrium | C56.9 | Malignant neoplasm of unspecified ovary |
|  |  |  | N76.0 | Acute vaginitis |
|  | C56.9 | Malignant neoplasm of unspecified ovary | R18.8 | Other ascites |
|  | O14.10 | Severe pre-eclampsia, unspecified trimester | Z23 | Encounter for immunization |
|  |  |  | D64.9 | Anemia, unspecified |
|  |  |  | J06.9 | Acute upper respiratory infection, unspecified |
|  |  |  | Z34.90 | Encounter for supervision of normal pregnancy, unspecified, unspecified trimester |
|  | O14.13 | Severe pre-eclampsia, third trimester | Z34.90 | Encounter for supervision of normal pregnancy, unspecified, unspecified trimester |
| 4 | C53.9 | Malignant neoplasm of cervix uteri, unspecified | N93.9 | Abnormal uterine and vaginal bleeding, unspecified |
|  | C56.9 | Malignant neoplasm of unspecified ovary | R18.0 | Malignant ascites |
|  | O14.10 | Severe pre-eclampsia, unspecified trimester | O60.03 | Preterm labor without delivery, third trimester |
|  |  |  | O32.9XX0 | Maternal care for malpresentation of fetus, unspecified, not applicable or unspecified |
|  |  |  | E11.8 | Type 2 diabetes mellitus with unspecified complications |
|  | O43.219 | Placenta accreta, unspecified trimester | O44.10 | Complete placenta previa with hemorrhage, unspecified trimester |
|  | O77.9 | Labor and delivery complicated by fetal stress, unspecified | K21.9 | Gastro-esophageal reflux disease without esophagitis |
|  | O99.42 | Diseases of the circulatory system complicating childbirth | I05.9 | Rheumatic mitral valve disease, unspecified |
| 5 | C18.9 | Malignant neoplasm of colon, unspecified | R10.9 | Unspecified abdominal pain |
|  | C53.9 | Malignant neoplasm of cervix uteri, unspecified | R50.9 | fever |
|  | C56.9 | Malignant neoplasm of unspecified ovary | F51.01 | Primary insomnia |
|  |  |  | I10 | Essential (primary) hypertension |
|  | D25.9 | Leiomyoma of uterus, unspecified | I80.209 | Phlebitis and thrombophlebitis of unspecified deep vessels of unspecified lower extremity |
|  |  |  | I11.9 | Hypertensive heart disease without heart failure |
|  | D49.5 | Neoplasm of unspecified behavior of other genitourinary organs | N92.0 | Excessive and frequent menstruation with regular cycle |
|  |  |  | I26.99 | Other pulmonary embolism without acute cor pulmonale |
|  | M32.9 | Systemic lupus erythematosus, unspecified | D68.61 | Antiphospholipid syndrome |
|  |  |  | I10 | Essential (primary) hypertension |
|  | O14.10 | Severe pre-eclampsia, unspecified trimester | O47.1 | False labor at or after 37 completed weeks of gestation |
|  |  |  | Z11.52 | Encounter for screening for COVID-19 |
|  |  |  | E78.5 | hyperlipidemia |
|  |  |  | N18.9 | Chronic kidney disease, unspecified |
|  |  |  | Z32.01 | Encounter for pregnancy test, result positive |
|  |  |  | O14.02 | Mild to moderate pre-eclampsia, second trimester |
|  |  |  | B02.9 | Zoster without complications |
|  | O32.1XX0 | Maternal care for breech presentation, not applicable or unspecified | O14.10 | Severe pre-eclampsia, unspecified trimester |
|  | O32.9XX0 | Maternal care for malpresentation of fetus, unspecified, not applicable or unspecified | J81.0 | Acute pulmonary edema |
|  | O34.29 | Maternal care due to uterine scar from other previous surgery | E04.9 | Nontoxic goiter, unspecified |
|  | O44.00 | Complete placenta previa NOS or without hemorrhage, unspecified trimester | O46.92 | Antepartum hemorrhage, unspecified, second trimester |
|  |  |  | O60.14X0 | Preterm labor third trimester with preterm delivery third trimester, not applicable or unspecified |
|  |  |  | Z02.79 | Encounter for issue of other medical certificate |
|  |  |  | O30.009 | Twin pregnancy, unspecified number of placenta and unspecified number of amniotic sacs, unspecified trimester |
|  | O45.90 | Premature separation of placenta, unspecified, unspecified trimester | O14.10 | Severe pre-eclampsia, unspecified trimester |
|  |  |  | O14.90 | Unspecified pre-eclampsia, unspecified trimester |
|  |  |  | O35.9XX0 | Maternal care for (suspected) fetal abnormality and damage, unspecified, not applicable or unspecified |
|  | O77.9 | Labor and delivery complicated by fetal stress, unspecified | R00.0 | tachycardia |
|  | O99.42 | Diseases of the circulatory system complicating childbirth | I50.9 | Heart failure, unspecified |
|  | R10.9 | Unspecified abdominal pain | O44.10 | Complete placenta previa with hemorrhage, unspecified trimester |
|  |  |  | Z39.2 | Encounter for routine postpartum follow-up |
|  |  |  | O86.81 | Puerperal septic thrombophlebitis |
|  |  |  | Z71.3 | Dietary counseling and surveillance |
|  |  |  | O24.419 | Gestational diabetes mellitus in pregnancy, unspecified control |
|  |  |  | O43.212 | Placenta accreta, second trimester |
|  | R57.1 | Hypovolemic shock | Z92.81 | Personal history of extracorporeal membrane oxygenation (ECMO) |
|  |  |  | O70.1 | Second degree perineal laceration during delivery |
|  |  |  | I27.0 | Primary pulmonary hypertension |
|  |  |  | O88.12 | Amniotic fluid embolism in childbirth |
|  | Z34.90 | Encounter for supervision of normal pregnancy, unspecified, unspecified trimester | O44.10 | Complete placenta previa with hemorrhage, unspecified trimester |
